# Emerging Health Concepts in the Probiotics Field: Streamlining the Definitions

**DOI:** 10.3389/fmicb.2019.01047

**Published:** 2019-05-21

**Authors:** Rebeca Martín, Philippe Langella

**Affiliations:** INRA, Commensal and Probiotics-Host Interactions Laboratory, Micalis Institute, AgroParisTech, Université Paris-Saclay, Jouy-en-Josas, France

**Keywords:** probiotics, live biotherapeutics, next-generation probiotics, prebiotics, synbiotics

## Introduction

**Figure 1:**
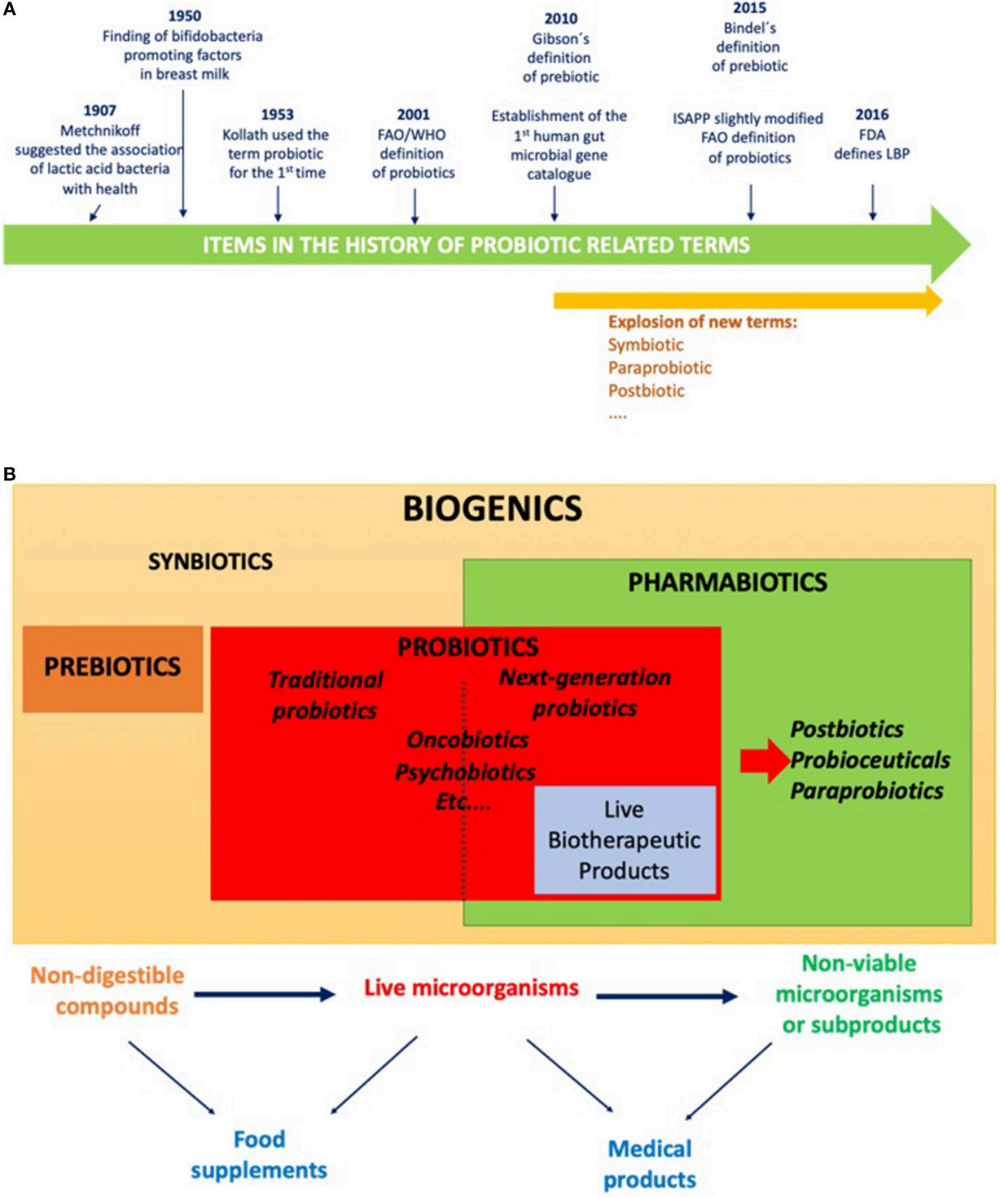
**(A)** Timeline of selected items in the history of probiotic related terms; **(B)** Scheme of the main terms related to probiotic field.

The concept of traditional probiotics was initially based on the observations of Elie Metchnikoff in 1907. He thus suggested that the regular consumption of fermented dairy products with lactic acid bacteria (LAB), such as yogurt, was associated with enhanced health and longevity in elderly Bulgarian people. Since then, the term probiotic has been always linked to beneficial bacteria for the host health, although its precise definition has evolved over the time (Figure 1A). Nowadays, thanks to the huge increase in the knowledge of the human gut microbiota and the importance of microbiota imbalances (dysbiosis) in several diseases and syndromes, probiotics can be used to restore the normal balance of the intestinal ecosystem as novel health promoting strategy. This new trend highlights the use of commensal bacteria as probiotics is the natural way to restore a healthy situation within different human ecosystems (intestinal, vaginal, skin…) opening the door to a new type of probiotics commonly called either Next-Generation Probiotics (NGPs) or Live Bio-Therapeutic Products (LBPs). Furthermore, the possibility of using non-viable bacteria, bacterial compounds, growth promoting substrates has opened the door to many new preventive therapy and options related to the field.

These new approaches have boosted the probiotic area during the last years with direct impacts on consumers and general public. As a result, nowadays a great number of new definitions have storm both the scientific and general world mixing up concepts and turning researchers, industrial partners, consumers, and regulators into stakeholders in the field that most of the times do not speak the same language.

## New Terms in the Field of Probiotics: When Definition Matters

Scientists, regulators and the food industry of the probiotic field share a common objective: to find high-quality profitable products based on high-quality scientific evidence-based studies that positively impact on the general population. Furthermore, for the commercialization of probiotics products, the regulators must find them reliable according to regulation and the consumers and healthcare providers should be well-informed and trust the evidences provided by the parts. The first is a challenge in terms of finding a common rule that is strict enough to protect potential consumers and could be adapted quickly to the constant progresses of the field. To achieve these objectives, the first step is that all the parts find a way to communicate and use the same terms in a consistent and meaningful way without misunderstandings, starting by basic concepts and definitions (Figure 1B). The more actual terms are summarized in Box 1.

Box 1 **Definitions related to probiotics field.**

**Probiotics:** live microorganisms that, when administered in adequate amounts, confer a health benefit to the host.

**Live Biotherapeutic Product (LBP):** a biological product that contains live organisms; is applicable to the prevention, treatment or cure of a disease or condition of human beings; and is not a vaccine.

**Next-Generation Probiotic (NGP):** live microorganisms identified on the basis of comparative microbiota analyses that, when administered in adequate amounts, confer a health benefit on the host.

**Prebiotic:** a non-digestible compound that, through its metabolization by microorganisms in the gut, modulates composition and/or activity of the gut microbiota, thus conferring a beneficial physiological effect on the host.

**Synbiotic/Symbiotic:** mixtures of probiotics and prebiotics that beneficially affect the host by improving the survival and implantation of live microbial dietary supplements in the gastrointestinal tract, by selectively stimulating the growth and/or by activating the metabolism of one or a limited number of health-promoting bacteria, thus improving host welfare.

**Pharmabiotics:** bacterial cells of human origin, or their products, with a proven pharmacological role in health or disease.

**Post-biotics:** non-viable bacterial products or metabolic products from microorganisms that have biologic activity in the host.

**Paraprobiotic/ghost probiotics/inactivated probiotics:** non-viable microbial cells (intact or broken) or crude cell extracts which when administered (orally or topically) in adequate amounts, confer a benefit to the human or animal consumer.

**Probioceuticals/probiotaceuticals:** probiotic derived factors.

**Biogenic:** products made by or of life forms including secretions and metabolites.

### Traditional vs. Next Generation Probiotic (NGP) Microorganisms

As stated above, probiotic definition has evolved since its creation and has been carefully analyzed by several experts. Nowadays, the most accepted definition by the scientific community is the one proposed by the expert panel convened by the International Scientific Association of Probiotics and Prebiotics (ISAPP) in 2014. It defines probiotics as “live microorganisms that, when administered in adequate amounts, confer a health benefit on the host” and proposed only a slight grammatical modification of the definition already proposed by the FAO/WHO in 2001. This definition itself differentiates commercial starter-cultures (administered as food processing elements) and commensal microorganisms (not administered but already present within the host) from probiotic bacteria that are administered to promote their health effects. Furthermore, it highlights the requirement to ingest live microorganisms. Although this definition has been kept mostly stable during the last 17 years, the transfer of the concept to the general public is still ongoing. The broad spectrum of the term has led several scientists to introduce subdivisions and the corresponding new terms considering the potential illness or system targeted. A subset of probiotic products may thus target disease, i.e., they will not address the general population but patients with a specific disease amenable to be treated by a particular probiotic product. In this sense, we can mention for instance either psychobiotics when the health benefit targets patients suffering from psychiatric illness (Dinan et al., 2013) or immunobiotics when the health improvement is targeting the mucosal immune level (Clancy, 2003).

Traditional probiotics have been isolated from many sources as gut and traditional fermented foods. They have been listed either as Generally Regarded as Safe (GRAS) at the strain level by the United States Food and Drug Administration (FDA) or as Qualified Presumption of Safety (QPS) at the species level by the European Food Safety Authority (EFSA). They mostly belong to a limited list of genera, basically, *Lactobacillus* spp. and *Bifidobacterium* spp. although there are also some members of *Bacillus* and *Escherichia coli* for bacteria and the yeast *Saccharomyces* among others. They have all a long history of use and their proved safety allowed thus their use as food or food supplements from a regulatory point of view. In contrast, NGPs have been recently isolated thanks to the new powerful tools to isolate, identify and even modify these commensal bacteria. They have been mostly identified based on comparative analysis of microbiota compositions between both healthy and unhealthy individuals and belong to diverse genera. They do not have a long history of safe use and their safety is not thus considered as proven. Both traditional and NGP fit to the classical definition of probiotics and could be daily administered to induce beneficial effects. Recently, the FDA has introduced the term of Live Biotherapeutic Product (LBP) that is “a biological product that contains live organisms; is applicable to the prevention, treatment or cure of a disease or condition of human beings; and is not a vaccine.” This term, sometimes proposed as a substitute of NGP, include the live biotherapeutic microorganism and the other ingredients that compose the final LBP. This is the reason why we strongly believe that the term LBP should not be systematically used to replace NGP. From our point of view, the concept of NGP is more extensive as it includes the microorganisms that will conform LBP but also those which are being analyzed and do not correspond to a potential defined product yet, including genetically modified bacteria and potential beneficial commensal bacteria.

### Prebiotics and Synbiotics/Symbiotics

The origin of the prebiotic concept started in the earlier 1950s with the discovery of growth promoting substances/factors in the human milk able to promote selective positive groups such as bifidobacteria. In 1995, the term prebiotic has been defined as “non-digestible food ingredients or substances that beneficially affect the host by selectively stimulating the growth and/or activity of one or a limited number of bacterial species already resident in the colon, and thus attempt to improve host health” (Gibson and Roberfroid, 1995). As in the case of the probiotics definition, this concept has evolved the last years. After several different definitions, Gibson has defined in 2010 that prebiotic is a “selectively fermented ingredient that results in specific changes in the composition and/or activity of the gastrointestinal microbiota, thus conferring benefit(s) upon host health” (Roberfroid et al., 2010).

The advances in the understanding of the interactions among the diet, the microbiota and the host have challenged this traditional concept of prebiotic, and, in contrast to the probiotic concept, there is still a debate in the scientific community to find a consensual definition. The open questions are: (i) the anatomical restriction to the gut; (ii) the requirement or not of fermentation; (iii) the restriction only to carbohydrates; and (iv) the requirement or not of microbiota modulation (possibility of having other direct positive effects) (Bindels et al., 2015). Taking this problematic into account, a ISAPP consensus panel have proposed in 2017 the most actual definition of prebiotic: “A substrate that is selectively utilized by host/commensal microorganisms conferring a health benefit” (Gibson et al., 2017).

Nowadays, the combination of probiotics and prebiotics in the same products is referred as synbiotics or symbiotics. They are defined as “mixtures of probiotics and prebiotics that beneficially affect the host by improving the survival and implantation of live microbial dietary supplements in the gastrointestinal tract, by selectively stimulating the growth and/or by activating the metabolism of one or a limited number of health-promoting bacteria, thus improving host welfare” (Gibson and Roberfroid, 1995). Nevertheless, the use of these terms is controversial as the word synbiotics is traditionally linked to symbiosis (any type of a close and long-term biological interaction between two different biological organisms) and the term symbiotic refers to a synergetic combination of pro and prebiotics where the second favored the first (Cencic and Chingwaru, 2010).

### Other Probiotic Related Terms

The term probiotics fits into a bigger category called pharmabiotics, which is defined as “bacterial cells of human origin, or their products, with a proven pharmacological role in health or disease” (Shanahan, 2009). This term is not restricted to live microorganisms and includes probiotics, bacteriocins, bacteriophages and bioactive molecules. In this category, we can include (i) post-biotics that are “non-viable bacterial products or metabolic products from microorganisms that have biologic activity in the host”(Patel and Denning, 2013); (ii) paraprobiotics (also called ghost or inactivated probiotics) that are “non-viable microbial cells (either intact or broken) or crude cell extracts which when administered (either orally or topically) in adequate amounts, confer a benefit on the human or animal consumer” (Taverniti and Guglielmetti, 2011); and (iii) probioceuticals/probiotaceuticals which defines probiotic derived factors such as reuterin from *Lactobacillus reuteri* (Howarth, 2010). The strong interest of these categories is the fact that these agents could provide their beneficial effects without the potential risk associated with the administration of live microorganisms. This point is very interesting regarding potential regulatory issues. Nevertheless, we consider that the use of these terms should be restricted to professional interchanges and we might try to minimize its use in translational activities to the general public until de general probiotic concept is generally well-assimilated for the non-specialist public.

## Future Directions

The increase in the knowledge in the field of probiotics suppose a unique scientific, translational and regulatory challenge. From the scientific point of view, the traditional researchers in the field of probiotics, classically microbiologists, have seen how the field has expanded including an increasing number of disciplines. This increased multidisciplinary is a great opportunity to progress but sometimes it could also trigger basic mistakes. In fact, only few laboratories involved in the probiotics domain count with all the skills required within their personnel. The solution for us could be quite simple: multidisciplinary research groups or consortiums to face this multidisciplinary field. There are not negligible actors as microbiologists are as essential as immunologists and physiologists.

From the regulatory point of view, there are big geographical differences that create difficulties in a global commercialization. Traditional probiotics are classified in different categories across countries (QPS for Europe and GRAS for USA) and cannot be used in most of health claims as the lactose intolerance prevention through yogurt ingestion is the only one health claim approved by European Food Safety Agency (EFSA). Furthermore, NGPs do not follow the same legislation worldwide. For instance, the use of live microorganisms that have not been traditionally used in food in Europe before 1997 is restricted and they should pass through hard regulatory process to be on the market in Europe either as a novel food with a health claim or as a drug. To launch on the market a non-traditional probiotic as a novel food, the regulatory process to follow is as complicated or even more as the one required for a drug even if the microorganism is a human commensal. This point, combined to the fact that most of the requirements are based on traditional probiotics, has already prompted us to propose a more appropriate framework for evaluation of microorganisms to be used as novel foods with a health claim in Europe (Miquel et al., 2015). On the other hand, Japan acts as a global market leader, where probiotics are considered as both foods and drugs and approves new food health claims on a regular basis. It is thus an evidence that regulatory framework should be updated to allow translational research and does not limit innovation being in parallel warrant of safety. Nevertheless, we are aware that geographical differences will subsist and that uniformity worldwide in a medium period of time is probably a dream.

From a translational point of view, the jungle of terms that we have exposed in this opinion paper is by itself an impediment in the transfer of the scientific knowledge. The best way to fight against lack of information related problems is to spread as much as possible rigorous scientific information. As researchers, one of our mission is to disseminate our research in a clear and non-disputable way. For this, we have to avoid using unnecessary new terms and slang and better define the key messages to communicate. We consider that it is not necessary to create a new word for every single advance. For instance, the word phagebiotics has been proposed to name bacteriophages used as pharmabiotics. When the general public is only starting to understand basic well-established terms such as probiotics, this strategy is only creating difficulties in the right interchange and do not provide advantages so in our opinion it should be avoided. Another problem is that outreach activities are not often well-recognized by the scientific community in terms of professional valorization. In a such competitive world such as the scientific one, this can be an obstacle. We strongly believe that new research strategies at all the levels should include a complete dissemination plan as a mandatory part of the research project proposals and properly valorize previously performed outreach activities.

In this perspective paper, our goal was to discuss all the emerging terms introduced due to the explosion of the knowledge in the probiotics area mainly during the last years. We aim to shed light on the differences among all the definitions in order to allow a clear and direct communication among all the concerned parts.

## Author Contributions

RM and PL wrote this opinion article.

### Conflict of Interest Statement

The authors declare that the research was conducted in the absence of any commercial or financial relationships that could be construed as a potential conflict of interest.
